# Real-Life Use of Posaconazole in Clinical Practice for Onco-Hematological Children: A National Survey by AIEOP Infectious Diseases Working Group

**DOI:** 10.3390/jof11110797

**Published:** 2025-11-07

**Authors:** Lorenzo Chiusaroli, Manuela Spadea, Cecilia Liberati, Maria Grazia Petris, Antonio Grasso, Francesco Baccelli, Maria Vittoria Micheletti, Pietro Gasperini, Maria Rosaria D’Amico, Katia Perruccio, Angelica Barone, Daniela Onofrillo, Paola Muggeo, Maura Faraci, Simona Rinieri, Ilaria Liguoro, Antonella Colombini, Francesca Trevisan, Nagua Giurici, Gianluca Boscarol, Letizia Pomponia Brescia, Alessia Pancaldi, Francesca Compagno, Alessandra Biffi, Daniele Donà, Simone Cesaro

**Affiliations:** 1 Division of Pediatric Infectious Diseases, Department for Women’s and Children’s Health, University Hospital of Padua, 35128 Padova, Italy; 2 Pediatric Onco-Hematology, Stem Cell Transplantation and Cellular Therapy Division, Regina Margherita Children’s Hospital, 10126 Turin, Italy; 3 Hematology and Oncology Unit, Department for Women’s and Children’s Health, University Hospital of Padua, 35128 Padova, Italy; 4 Pediatric Hematology and Oncology, IRCCS Azienda Ospedaliero-Universitaria di Bologna, 40138 Bologna, Italy; 5 Pediatric Hematology Oncology, Bone Marrow Transplant, Azienda Ospedaliera Universitaria Pisana, 56121 Pisa, Italy; 6 Department of Pediatrics, Azienda Unità Sanitaria Locale di Rimini, 47921 Rimini, Italy; 7 UOC TCE and Cellular Therapies, Department of Pediatric Hematology-Oncology and Cellular Therapies, Santobono-Pausilipon, 80123 Naples, Italy; 8 Pediatric Oncology Hematology, Mother and Child Health Department, Santa Maria Della Misericordia Hospital, 06100 Perugia, Italy; 9 Pediatria e Oncoematologia, Azienda Ospedaliero-Universitaria di Parma, 43126 Parma, Italy; 10 Pediatric Oncology Hematology, Civil Hospital Santo Spirito, 65124 Pescara, Italy; 11 Pediatric Oncology and Hematology, University Hospital of Policlinico, 70124 Bari, Italy; 12 Hematopoietic Stem Cell Transplantation Unit, Department of Hematology and Oncology, IRCCS Institute G. Gaslini, 16147 Genoa, Italy; 13 DH Pediatric Oncology Hematology, University Hospital S. Anna Ferrara, 44121 Ferrara, Italy; 14 Division of Pediatrics, University Hospital of Udine, 33100 Udine, Italy; 15 Department of Pediatrics, Fondazione MBBM-Ospedale San Gerardo, 20900 Monza, Italy; 16 Pediatric Onco-Hematology Unit-AOU Meyer Firenze, 50139 Florence, Italy; 17 UO Pediatric Hemato-Oncology, Institute for Maternal and Child Health (I.R.C.C.S.) Burlo Garofolo, 34137 Trieste, Italy; 18 Department of Pediatrics, Central Teaching Hospital Bolzano, 39100 Bolzano, Italy; 19 Hemato-Oncology Unit, SS Annunziata Hospital, 74100 Taranto, Italy; 20 Pediatric Oncology and Hematology Unit, Department of Medical and Surgical Sciences for Mother Children and Adults, University of Modena and Reggio Emilia, 41124 Modena, Italy; 21 Pediatric Hematology/Oncology Unit, Fondazione IRCCS Policlinico San Matteo, 27100 Pavia, Italy; 22 Pediatric Hematology Oncology, Department of Mother and Child, Azienda Ospedaliera Universitaria Integrata, 37126 Verona, Italy

**Keywords:** posaconazole, fungal infections, survey, children

## Abstract

Background: Posaconazole is an antifungal medication used to treat invasive fungal infections (IFI) in pediatric onco-hematological patients. Its approval for pediatric use was recent, and limitations still apply. Despite limited data, the safety and efficacy profile appear generally favorable in children. This study describes how posaconazole is used across centers affiliated with the Associazione Italiana Ematologia e Oncologia Pediatrica (AIEOP). Methods: A national survey was conducted among physicians within the AIEOP network to evaluate current use of posaconazole in pediatric cancer patients, including those undergoing hematopoietic stem cell transplantation (HSCT). A 25-item web questionnaire was developed and distributed in June 2024. Data analysis involved descriptive statistics. Results: Twenty-one of thirty-one centers (68%) responded, reporting availability of various posaconazole formulations: oral suspension (76%), delayed-release tablets (95%), and intravenous solution (14%). Posaconazole was primarily used for prophylaxis in patients with acute lymphoblastic leukemia (ALL, 38%), acute myeloid leukemia (AML, 38%), and aplastic anemia (19%). It was also used as secondary prophylaxis against previous possible or probable IFI or as salvage therapy for probable or confirmed aspergillosis or mucormycosis, often combined with other treatments. Drug plasma level monitoring was common but varied in scheduling across centers. Most centers (74%) discontinued posaconazole if adverse events suspected drug–drug interactions, such as with vincristine. Conclusions: Posaconazole is widely used in AIEOP centers, though application varies significantly. This variability emphasizes the need for prospective studies to better define indications, dosing, and monitoring protocols for pediatric use of this antifungal.

## 1. Background

Children with cancer are at increased risk for several opportunistic infections, among which invasive fungal infections (IFI) have significant morbidity and mortality. Azoles are the most tolerated agents for treatment and prophylaxis, offering a good efficacy profile and limited side effects [1,2]. Among azoles, posaconazole exhibits activity against yeasts and molds, including *Mucorales.* [3] In 2013, the Food and Drug Administration (FDA) approved a delayed-release (DR) tablet formulation for patients 13 years of age and older for the prophylaxis of invasive *Aspergillus* and *Candida* infections in patients at high risk for fungal infections [4].

Further, in 2021, DR tables and intravenous (IV) solution received the authorization for patients aged >2 years old and weighing more than 40 kgs by the European Medicines Agency (EMA) and only in 2024 by Agenzia Italiana del Farmaco (AIFA) [5].

The formulations are not interchangeable, and achieving the correct threshold level can be challenging in the pediatric population due to highly variable bioavailability and depending on gastric pH, food, mucositis, diarrhea, and medications [6,7].

However, the use of these compounds remains controversial, particularly in prophylaxis, because of potential interactions with the chemotherapeutic protocol [8].

This study aims to analyze clinical practices related to the use of posaconazole in centers affiliated with the Associazione Italiana Ematologia e Oncologia Pediatrica (AIEOP) in the pediatric oncologic–hematologic population.

## 2. Methods

We conducted a comprehensive national survey during June and July 2024 among physicians affiliated with the AIEOP network, which comprises 31 centers, including 21 centers specializing in hematopoietic stem cell transplantation (HSCT). The purpose of this survey was to evaluate the current utilization of posaconazole in pediatric oncological patients across various Italian centers, including those performing HSCT. An ad hoc developed 25-item web-based questionnaire was disseminated to all AIEOP centers in June 2024. Each item in the questionnaire featured multiple-choice responses, most of which permitted multiple selections. The complete questionnaire and the responses received are summarized in Table 1.

Data were analyzed by descriptive statistics. The primary outcomes examined were efficacy when used in prophylaxis or treatment, formulation and dosage, therapeutic drug monitoring (TDM) and drug–drug interaction (DDI).

## 3. Results

The survey was distributed to 31 Italian hospitals and children’s hospitals. A total of 21 hematologists, pediatric hematologists, or pediatric infectious disease specialists with experience in treating invasive fungal infections (IFIs) responded. Most Italian regions (13 out of 20) participated in the survey, allowing for an almost nationwide assessment.

### 3.1. Formulations and Dosage

**Figure 1:**
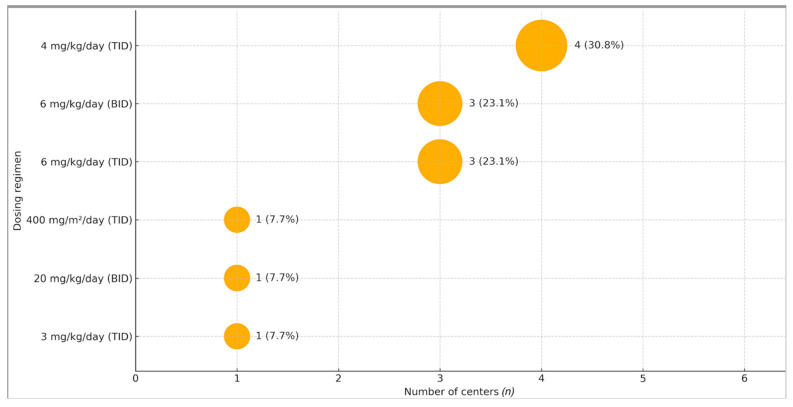
Dosage schemes adopted among the centers according to our Survey. TID: three times daily. BID: twice daily. Note: 8/21 centers did not provide the exact dosage and are excluded. Percentages are calculated among the 13 reporting centers.

Posaconazole was available at all centers participating in the survey (21/21). The formulations available varied: most centers could prescribe oral suspension (16/21, 76%), DR tablets (20/21, 95%), and a few centers could provide IV solution (3/21, 14%). Centers more frequently used the oral suspension in patients younger than eight years (17/21, 81%), while DR tablets were used by a minority in this age group (19). In contrast, in older children aged 8–12 years, oral suspension and DR tablets were administered in 52% (11/21) and 62% (13/21) of centers, respectively. None of the centers used IV solution. Regarding posology, different administration schedules were adopted for oral suspension (Figure 1): 4 mg/kg three times daily (4/21 centers), 6 mg/kg three times daily (2/21), 6 mg/kg twice daily (3/21), 3 mg/kg three times daily (1/21), 20 mg/kg divided into two doses (1/21), and 400 mg/m^2^ divided into three doses (1/21). Eight centers did not respond to this question. For DR tablets, 10 centers administered dosage stratified by weight (<10 kg, 10–20 kg, >20 kg), based on published literature; another 10 centers dosed according to mg/kg without specifying the exact amount.

### 3.2. Use of Posaconazole for Primary Prophylaxis

Based on our survey results, 13 out of 21 (62%) centers use posaconazole for antifungal prophylaxis: 8 out of 21 (38%) for high-risk acute lymphoblastic leukemia (ALL) patients with high-risk leukemia, 7 out of 21 (33%) for relapse ALL, and 11 out of 21 (52%) for acute myeloid leukemia (AML). Nine centers (43%) performed HSCT, and five of those used posaconazole as primary prophylaxis in allogeneic HSCT. For aplastic anemia, posaconazole was used in only 4 out of 21 institutes. Eight centers did not administer posaconazole for prophylaxis due to various reasons, including rare mold cases in local epidemiology (3/21), lack of confidence in the drug (1/21), pharmacy restrictions (1/21), and other unspecified reasons (3/21).

Furthermore, Posaconazole was used as secondary prophylaxis for previous possible and probable IFI in 76% (16/21) and 81% (17/21) of cases, respectively. It was administered in 3 out of 6 centers for allogeneic HSCT and in 2 out of 6 centers for autologous HSCT as secondary prophylaxis.

### 3.3. Use of Posaconazole for Treatment

In our survey, five out of 21 centers (24%) did not empirically administer posaconazole, even in cases of prior IFI. Instead, clinicians tended to prescribe posaconazole for possible or probable IFI in patients with (9/21, 43%) and without (8/21, 38%) a previous history of IFI, often as salvage therapy.

In patients on posaconazole prophylaxis who develop fever, approximately half of the centers discontinue prophylaxis and start empirical antifungal therapy (11/21, 55%), while 38% (8/21) repeat the diagnostic check-up and decide based on the results, and 9% (2/21) continue prophylaxis with posaconazole and evaluate according to the patient’s clinical condition.

**Figure 2:**
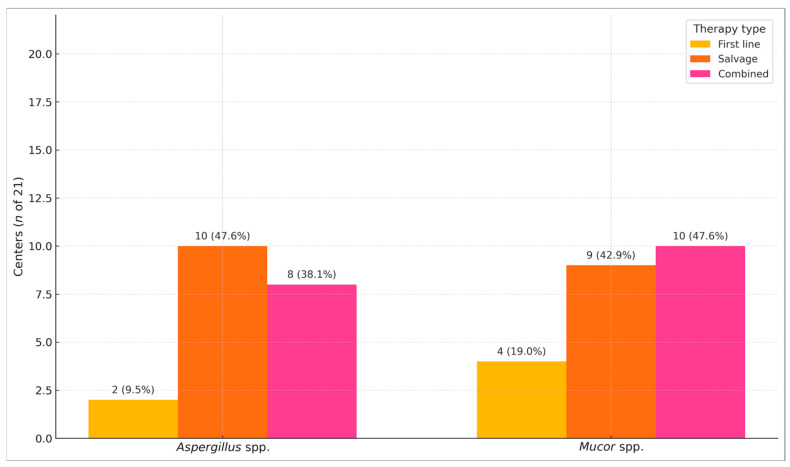
Number of centers where Posaconazole is used for treatment. For this item, the center could select one or more options. Combined therapy refers to combination with other antifungal agents.

For IFI due to *Aspergillus* spp., posaconazole was used as first-line therapy in 2/21 (9%) of centers, as salvage therapy in 10/21 (48%), and as combined therapy in 8/21 (38%) centers. Regarding *Mucor* spp. infection, posaconazole was adopted as first-line therapy in 4/21 (9%) of centers, as salvage therapy in 9/21 (43%), and as part of combined therapy with other antifungal agents in 10/21 (48%) centers (Figure 2).

### 3.4. Therapeutic Drug Monitoring and Interactions

The centers outlined the following strategies to monitor TDM of posaconazole: blood sampling within five days of starting posaconazole was practiced in 4/21 (19%) centers, between five and seven days in 10/21 (48%), and after seven days in 4/21 (19%) centers. For TDM assessment after reaching the threshold level, most centers (10/21, 48%) scheduled checks once every two weeks, 4/21 (19%) did so weekly, and 1/21 (5%) opted for monthly assessments. One center (1/21, 5%) reported no specific timing policy, choosing based on clinical response and signs of toxicity. Managing interactions with vincristine proves challenging, with most centers preferring to stop prophylaxis with posaconazole (10/21, 48%) or switch to another antifungal (4/21, 19%). Only five centers (24%) continue using posaconazole while monitoring its threshold levels.

## 4. Discussion

To our knowledge, this is the first national survey describing how Italian pediatric onco-hematology centers currently use posaconazole and how these practices align with the limited pediatric evidence base. The overall picture shows broad familiarity but heterogeneous application, a pattern consistent with international guidance that is either adult-focused, cautious in children, or explicitly highlights evidence gaps [9,10,11,12].

This variability is influenced by physician confidence, the local epidemiology of invasive fungal infections, and the absence of a standardized consensus on posaconazole use in the pediatric population. The limited number of studies, challenges in achieving both prophylactic and therapeutic plasma levels, and the potential for DDI during chemotherapy protocols are key factors influencing its use.

Not surprisingly, routine primary prophylaxis with posaconazole is adopted by only a subset of Italian centers (13/21), mirroring the guarded stance of pediatric-specific guidelines. The European Conference on Infections in Leukemia (ECIL) 8 pediatric guidance recognizes feasibility and safety but stops short of strong, universal pediatric recommendations because comparative pediatric data versus other azoles/echinocandins are sparse, and Infectious Diseases Society of America (IDSA) guidance is similarly conservative for children [9,10].

Where adopted, our respondents cite perceived safety and convenience, aligning with series that report low breakthrough infection and acceptable tolerability compared with alternative agents [13].

Secondary prophylaxis is used by most centers (17/21), yet high-quality pediatric data supporting this indication are scant, and international guidance calls for individualized use pending better evidence [11].

The more assertive positions observed in adult AML/allogenic-HCST populations cannot be directly extrapolated to younger children, a point echoed in ECIL 2025 [14].

Consistent with international guidelines, Italian centers rarely use posaconazole for empirical therapy in febrile neutropenia or for suspected *Aspergillus* or *Mucor* spp.; first-line use is not recommended in children [11].

By contrast, its targeted role against mucormycosis is recognized: when liposomal amphotericin B cannot be given or as step-down/continuation therapy, DR tablets or (IV) formulations are preferred for more predictable exposure [15].

This mirrors pediatric experiences from registry and cohort studies that emphasize combined medical–surgical approaches and, when used, azole–polyene combinations on a case-by-case basis, although pediatric-specific combination data remain limited [16].

Practice heterogeneity in Italy closely tracks formulation-driven pharmacokinetic (PK) variability. The oral suspension has erratic bioavailability (affected by gastric pH and motility), making dosing particularly challenging in younger children and during mucositis or proton-pump inhibitor use [17,18,19].

DR tablets and IV formulations offer more stable exposures, but our survey indicates no IV use and wide variability in initial dosing regimens (six distinct approaches across centers, see Table 1), reflecting uncertainty about optimal pediatric dosing. Available pediatric PK/TDM studies show that thrice-daily oral-suspension dosing (e.g., 18 mg/kg TID) often fails to achieve target troughs in a substantial proportion of patients [17]. This issue aligns with our survey findings, which highlight the diverse dosing strategies clinicians use in real-world practice.

International guidance supports TDM to optimize efficacy and minimize toxicity, with pragmatic trough targets of >0.7 mg/L for prophylaxis and >1.0 mg/L for treatment [20,21].

The Society of Infectious Diseases Pharmacists (SIDP) suggests timing troughs at steady state—approximately day 5 with a loading dose and approximately day 7 without [22].

Our respondents reported divergent TDM schedules and thresholds, underscoring the absence of a standardized pediatric algorithm and the need for consensus on loading, target attainment, and dose-adjustment rules [23].

Finally, the survey corroborates real-world caution with azole–vincristine coadministration in ALL protocols. Case reports and small series associate concurrent azoles (including posaconazole) with increased neurotoxicity, notably autonomic neuropathy and constipation, likely via CYP3A4 inhibition [7,24].

Several authors advise interrupting azole prophylaxis for a short interval around vincristine dosing when alternatives are not feasible. This safety signal is well recognized internationally and likely contributes to discontinuations observed in our cohort (in most centers, occurring when adverse events arise) [25,26,27].

In conclusion, our survey emphasizes the challenges associated with the use of posaconazole, including limitations in clinical placement, complex administration regimens, and the necessity for therapeutic drug monitoring. Furthermore, its use has been restricted to oral suspension and delayed-release (DR) tablet formulations, despite the availability of the intravenous (IV) formulation. The lack of randomized trials and, consequently, clear guideline recommendations restrict its widespread adoption, and our survey also clearly shows clinicians’ uncertainty regarding its optimal use in real-world settings. We summarize in Table 2 the ECIL recommendations for pediatric use of posaconazole, compare them with the survey results, and outline potential future directions for this drug. Additionally, barriers to using posaconazole in children include difficulties in administering oral antifungal prophylaxis to very young children and in cases of painful mucositis or enteritis after chemotherapy. Moreover, unlike other prophylactic measures such as those for *Pneumocystis*, posaconazole and other antifungals are not explicitly included in treatment protocols, leaving their use at the discretion of individual centers.

Looking ahead, clearer pediatric recommendations will require better data: prospective, adequately powered studies of prophylaxis in defined high-risk groups; standardized, exposure-guided dosing anchored by pragmatic TDM; and pediatric-specific evaluations of combination therapy and DDI-mitigation strategies. The recent approval of posaconazole for pediatric use by European and national regulatory agencies, along with the introduction of a powder for delayed release oral suspension, may lower logistical barriers and catalyze such studies, but coordinated multicenter efforts—nationally and in step with ECIL, IDSA, and ECMM priorities—are still essential to move from heterogeneous practice to evidence-based standardization [9,10,11,14,28].

## Figures and Tables

**Table 1:** Complete Survey questionnaire and responses.

Question	Centers Responses (*n* = 21)
***Which formulations of Posaconazole are available in your institution?***	
Oral suspension	16/21
Delayed release tablets	20/21
Intravenous solution	3/21
***Which formulations are used more frequent?***	
Oral suspension	17/21
Delayed release tablets	4/21
Intravenous solution	0/21
***Which posology do you use for oral suspension?***	
4 mg/kg three times daily	4/21
6 mg/kg three times daily	3/21
6 mg/kg twice times daily	3/21
3 mg/kg three times daily	1/21
20 mg/kg divided in twice	1/21
400 mg/m^2^ divided in three doses	1/21
***Which posology do you use with delayed release tablets?***	
Stratifying for weight ^#^	10/21
Mg/kg, not specify the dosage	10/21
***Do you use the posaconazole for the antifungal prophylaxis?***	
Yes	13/21
No	8/21
Local Epidemiology	3/2
Not allowed by pharmacy	1/21
Not experience with the drug	1/21
Not specified	3/21
***For which disease do you use?***	
ALL high-risk	8/21
Relapse ALL	7/21
AML	11/21
Allogenic HSCT	5/21
Autologous HSCT	0/21
Aplastic anemia	4/21
***Do you use, or have you used Posaconazole for secondary prophylaxis after a Possible or Probable invasive fungal infection?***	
Possible: Yes	16/21
Probable: Yes	17/21
***Do you use, or have you used Posaconazole as empirical antifungal treatment in patients with fever, neutropenia and with a history of fungal infection?***	
Yes	5/21
No	16/21
***Do you use, or have you used Posaconazole as an antifungal treatment in patients with invasive fungal infection Possible and Probable with or without previous fungal infection?***	
Possible without previous infection: Yes, not as first line therapy	5/21
Probable without previous infection: Yes, not as first line therapy	8/21
Possible with previous infection:	
Yes, as first line therapy	3/21
Yes, not as first line therapy	6/21
Probable without previous infection:	
Yes, as first line therapy	3/21
Yes, not as first line therapy	6/21
***In the patient with persistent fever and neutropenia, in prophylaxis with Posaconazole, how do you behave if the patient is febrile after 72–96 h of broad-spectrum antibiotic?***	
Discontinue the prophylaxis and start empirical antifungal therapy	11/21
Repeat the diagnostic check-up and decide based on this	8/21
Maintain prophylaxis with Posaconazole and evaluating according to the patient’s clinical conditions	2/21
***How do you consider the use of posaconazole in Aspergillus* spp. *infections?***	2/21
First line therapy	10/21
Salvage therapy or second line	8/21
Combined therapy	
***How do you consider the use of posaconazole in Mucor* spp. *infections?***	4/21
First line therapy	9/21
Salvage therapy or second line	10/21
Combined therapy	
***When do you check the first serum level after starting Posaconazole?***	
Within five days	4/21
Between 5 and 7 days	10/21
After 7 days	4/21
***How often do you repeat the serum level (TDM) of Posaconazole after reaching the optimal level?***	
Once every two weeks	10/21
Once weekly	4/21
Once monthly	1/21
Tailored strategy according to the patient	1/21
***How do you manage the posaconazole/vincristine interactions?***	
Discontinue posaconazole	10/21
Switch to another antifungal drug	4/21
Continue using posaconazole while monitoring its threshold level	5/21

^#^ We considered <10 kg, 10–20 kg, >20 kg dosing according to the current guidelines. AML: Acute Myeloid Leukemia. ALL: Acute Lymphoblastic Leukemia. TDM: Therapeutic drug monitoring. HSCT: Hematopoietic Stem Cell Transplantation.

**Table 2:** Posaconazole Survey findings compared with the European Conference on Infections in Leukemia (ECIL) recommendations.

Domain	Recommendation	Survey Findings	Appraisal	Comments	Implication for Practice
**Primary prophylaxis—AML**	Strongly supported in adults and pediatric	Used in 11/21 (52%) centers	Partially aligned	Reflect clinicians’ preferences other routinely used, easily available and manageable drugs	Standardize indication in national pediatric specific guidelines
**Primary prophylaxis—ALL (including relapse)**	Generally recommended in selected children with first line ALL (see the text); universally recommended in relapse	Used in 8/21 (38%) (high-risk) and 7/21 (33%) (relapse)	Partially aligned	Reflect clinicians’ preferences other routinely used, easily available and manageable drugs	Standardize indication in national pediatric specific guidelines
**Primary prophylaxis–allogeneic HSCT**	Supported in adults and pediatric, especially pre-engraftment and GVHD	Used in 5/9 HSCT centers	Partially aligned	Reflect clinicians’ preferences other routinely used, easily available and manageable drugs	
**Secondary prophylaxis**	Case-by-case in pediatrics	Used in 16/21 (possible); 17/21 (probable)	Generally consistent, evidence base limited		
**Formulations**	Prefer DR tablets/IV for predictable exposure; avoid suspension when absorption impaired	DR available 20/21 (95%), IV 3/21 (14%); suspension widely used in <8 y	Mixed; IV access appears constrained	Reflect limited resources (unavailability of the IV formulation in Italy)	Work with pharmacy/health authorities to ensure DR tablet and IV access supply nationwide
**Dosing—DR tablets**	Weight-banded per label/guidelines	10/21 weight-banded; 10/21 “mg/kg (unspecified)”	Heterogeneous; many unspecified	Reflects heterogeneity of dosing guidelines	Standardize dosing in national pediatric specific guidelines
**Dosing—Suspension**	Erratic PK; avoid if poor GI absorption; if used, exposure-guided	Six different schedules reported	Highly variable	Reflects heterogeneity of dosing guidelines	Standardize dosing in national pediatric specific guidelines
**TDM—first level**	Trough at steady state (~day 5 with load; ~day 7 without)	19% ≤ 5 d, 48% 5–7 d, 19% > 7 d	Mostly aligned, some late	Reflect limited resources (TDM not available in the same hospital)	Ensure funding and lab access for timely assays
**TDM—maintenance**	Recheck after changes/interactions; pragmatic q1–2 weeks in high-risk	48% q2w, 19% weekly, 5% monthly, 5% tailored	Reasonable overall, monthly may be too sparse in unstable settings		
**Vincristine DDI**	Prefer to avoid co-administration; consider temporary interruption or switch	48% stop, 19% switch, 24% continue with TDM	~67% aligned; 24% potentially risky	Reflect center-specific policy	Build a prospective registry on azole–vincristine management

The table summarizes the European Conference on Infections in Leukemia (ECIL) 8 and 10 recommendations concerning the use of posaconazole in children, compares them with the survey results, and outlines potential future perspectives for this compound. AML: acute myeloid leukemia; ALL: acute lymphoblastic leukemia; HCT: hematological stem cell transplantation; DR: Delayed release; PK: pharmacokinetic; GI: gastrointestinal; TDM: Therapeutic drug monitoring; q2w: once every 2 weeks; DDI: drug–drug interaction.

## Data Availability

The original contributions presented in this study are included in the article. Further inquiries can be directed to the corresponding author.
